# Challenges, current status and emerging strategies in the development of rapidly dissolving FDM 3D-printed tablets: An overview and commentary

**DOI:** 10.5599/admet.1622

**Published:** 2023-01-01

**Authors:** Abu T.M. Serajuddin

**Affiliations:** Department of Pharmaceutical Sciences, College of Pharmacy and Health Sciences, St. John’s University, 8000 Utopia Parkway, Queens, NY 11439, USA; serajuda@stjohns.edu

**Keywords:** 3D printing, fused deposition modeling, hot melt extrusion, 3D-printed tablet, drug release, rapid dissolution

## Abstract

Since the approval of a 3D-printed tablet by the FDA in 2015 for marketing, there has been a great interest in 3D printing in the pharmaceutical field for the development of personalized and on-demand medications. Among various 3D printing methods explored for the development of oral solid dosage form like tablet, the fused deposition modeling (FDM) 3D-printing, where the drug-polymer mixtures are first converted into filaments by hot melt extrusion (HME) and then the filaments are printed into tablets using 3D printers by applying computer-aided design principles, has emerged as the most attractive option. However, no FDM 3D-printed tablets have yet been marketed as the technology faces many challenges, such as limited availability of pharmaceutical-grade polymers that can be printed into tablets, low drug-polymer miscibility, the need for high temperature for HME and 3D-printing, and slow drug release rates from tablets. These challenges are discussed in this article with a special focus on drug release rates since FDM 3D-printing usually leads to the preparation of slow-release tablets while the rapid release from dosage forms is often desired for optimal therapeutic outcomes of new drug candidates. Pros and cons of various strategies for the development of rapidly dissolving FDM 3D-printed tablets reported in the literature are reviewed. Finally, two case studies on emerging strategies for the development of rapidly dissolving FDM 3D-printed tablets are presented, where one outlines a systematic approach for formulating rapidly dissolving tablets, and the other describes a novel strategy to increase dissolution rates of drugs from FDM 3D-printed tablets, which at the same time can also increase drug-polymer miscibility and printability of tablets and lower processing temperatures. Thus, this overview and commentary discusses various issues involving the formulation of rapidly dissolving FDM 3D-printed tablets and provides guidance for the development of commercially viable products.

## Introduction

Three-dimensional (3D) printing, where materials are deposited layer by layer to form solid objects, has gained much interest in the pharmaceutical field as it can deliver personalized and on-demand medications, such as tablets, to patients for better therapy [1]. 3D-printing is essentially a form of additive manufacturing (AM) that is extensively used in many different industries [2]. The interest in 3D printing in the pharmaceutical field has been most intense since the approval of a 3D-printed tablet, Spiritam®, by the US Food and Drug Administration in 2015 [3], which is evident from the exponential increase in the number of publications on 3D printing of pharmaceutical products in recent years [4, 5]. Various opportunities provided by 3D printing technology in the pharmaceutical field and different challenges currently facing its application in the development of drug products have been extensively reviewed in the literature [6-12].

Several different 3D-printing techniques, such as binder jet printing, fused deposition modeling (FDM), semisolid extrusion (SSE), selective laser sintering (SLS), and stereolithography (SLA), have been employed for the manufacture of tablets [1,11]. Among these techniques, fused deposition modeling (FDM) 3D-printing is the most popular and widely used for the development of solid dosage forms like tablets and pellets. In this method, drug-loaded filaments of thermoplastic polymers are produced by hot melt extrusion (HME) and the filaments are then printed into tablets using 3D printers by applying computer-aided design (CAD) principles [13,14]. Hot melt extruders used for the preparation of filaments are now commonly available in academia and the pharmaceutical industry for many different applications and 3D printers are relatively cheaply available in the market for the printing of filaments into tablets, both of which contributed to the rapid growth of the FDM 3D-printing technology. It is anticipated that, in the future, the filaments can be mass-produced in the pharmaceutical industry or at various pharmaceutical distribution sites and then sent to clinics, retail pharmacies, hospitals, nursing homes, etc., for printing into individualized medications as per the needs of the patients. Such personalized therapy can mitigate some of the variability in patients’ responses to medications observed due to differences in their genetic compositions, gender, age, body weight, disease state, and so forth. The FDM 3D printing will be especially suitable for clinical testing of new drug candidates where the dose is often unknown at the outset of clinical studies and may need to be changed or modified depending on clinical observations on patients. Under such circumstances, clinical studies may be accelerated as new doses may be printed using the same filaments without having to wait for the development of new formulations. Additionally, FDM 3D printing has the potential to provide multiple drugs in the same tablet by printing different drugs layer by layer, side by side or in different compartments [15,16]. The doses may also be individualized in such polypills.

## Objectives of this report

Despite its early promises and relative simplicity, no products based on the FDM 3D-printing technology have yet been approved by regulatory agencies and marketed by pharmaceutical companies. This is because FDM 3D-printing is a relatively new technology that faces many developmental and technical challenges. The objectives of the present Overview and Commentary are to critically analyze these challenges, discuss what progress has been made towards resolving them, and present emerging strategies that may lead to the resolution of most of these problems leading to the development of clinically and commercially viable FDM 3D-printed tablets. One special focus will be on the development of rapidly dissolving tablets since FDM 3D-printing mostly produces tablets with slow and incomplete drug release, while tablets with rapid drug release rates are often needed for optimal clinical efficacy in humans. The importance of rapid drug release for the successful therapeutic outcome of new drug candidates will be discussed based on the Biopharmaceutical Risk Assessment Roadmap (BioRAM) developed by an international panel of experts [17,18], and the pros and cons of several strategies for the development of rapidly dissolving 3D-printed tablets reported in the literature will be reviewed. Finally, two case studies will be presented where one of them will provide a systematic approach for the development of FDM 3D-printed tablets for rapid drug release based on the work by Solanki et al. [19], and the other will provide, based on two recent publications by Patel and Serajuddin [20,21], a multi-pronged strategy to not only increase drug release rates from printed tablets but at the same time to also enhance drug-polymer miscibility, increase printability of polymers, and reduce processing temperatures of filaments and tablets.

## Challenges in the development of FDM 3D-printed tablets

As outlined by Solanki et al. [19] and Patel and Serajuddin [20,21], the FDM 3D-printing technology faces several major challenges that must be addressed before it can progress from the current scientific exploratory phase to a mature technology for the development and manufacture of viable and commercially successful drug products. Some of these challenges are:

- limited availability of pharmaceutical-grade polymers that are suitable for FDM 3D-printing,
- low drug drug-polymer miscibility,
- the need for high temperatures for melt extrusion and FDM 3D-printing where drug, polymer or both may degrade, and
- slow and incomplete drug release from tablets.

**Figure 1.**
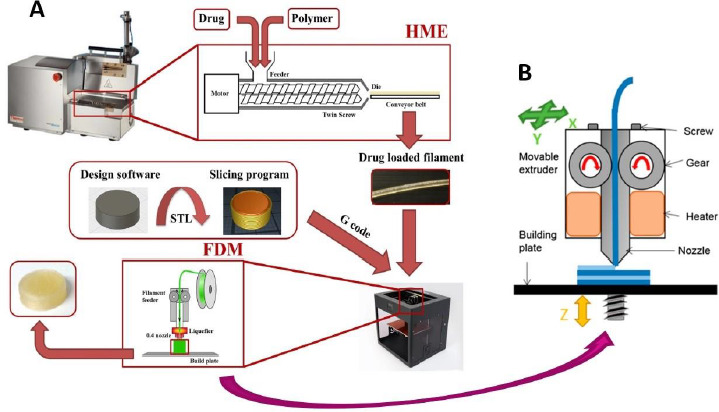
(A) Illustration of melt extrusion of filaments and 3D-printing of tablets during the FDM 3D-printing process. (B) Printing gear, nozzle, and printing plate are shown with an expanded scale. Adapted from Ilyes et al. [22] with permission.

The above challenges in the development of FDM 3D-printed tablets are discussed below using Fig. 1, where different steps involved in the preparation of FDM 3D-printed tablets are given in Fig. 1A, while Fig. 1B shows the printing process on an expanded scale:

### Limited availability of pharmaceutical-grade polymers

As shown in Fig. 1A, the extrusion of drug-polymer mixtures through a hot melt extruder into filaments is the first major step in the FDM 3D printing process. For this purpose, it is essential that the polymers used are extrudable. However, melt extrusion is a relatively new application of polymers in the pharmaceutical industry, and most polymers currently used for the formulation of conventional tablets were originally developed and introduced by their manufacturers for other pharmaceutical applications like diluent, binder, disintegrant, drug release modifier, coating agent, etc. As such, they may not have suitable attributes, such as glass transition temperature (*T*_g_), melt viscosity, thermal stability, etc., for HME. Even when certain polymers and drug-polymer mixtures are extrudable, the filaments produced may not be printable. Various issues with the feeding of filaments into 3D printers and then printing as tablets have been reported in the literature [23,24]. As shown in Fig. 1B, the filaments are pushed through the printing nozzles by using gears, where they may break if they are too brittle or may not pass through the nozzle at all if they are too soft. Therefore, the optimal flexibility of a filament is essential for its printing into a tablet. Additionally, once the filaments are fed into the printer using the gear, they are further heated to attain certain optimum melt viscosities such that the molten materials are streamed through the printer head and deposited on the building plate layer by layer to form tablets [25]. The layers must coalesce with each other, and they must also harden rapidly enough that the surface, shape, and internal structures remain intact as the tablets are built by depositing one layer over the other until the printing is completed [26]. All these attributes of filaments and tablets often depend on the properties of the polymers used. For this reason, Henry et al. [27] cautioned that despite much academic research and many proof-of-concept studies, a more thorough understanding of polymer properties and the interplay between polymers and processing parameters are needed before the FDM 3D-printing technique can be fully implemented in the development of personalized medicine. Currently, the availability of pharmaceutical-grade polymers that are extrudable and printable remains a major limitation in the progress of FDM 3D printing technology.

### Lack of drug-polymer miscibility

Ensuring drug-polymer miscibility is another major challenge in the development of FDM 3D-printed tablets. Although it may not be a major issue for water-soluble drugs since they may dissolve rapidly even if they remain crystalline and phase-separated from polymers, one must consider that approximately 3 out of 4 chemical entities under development as drug candidates are poorly water-soluble and may fall into Class II or IV of the Biopharmaceutical Classification System (BCS) [28]. This is reflected in the types of drugs used for 3D printing. In a survey of literature for 37 drugs used for FDM 3D printing, Pereira et al. (29) observed that 28 (74 %) belonged to BCS class II or IV, indicating possible solubility and dissolution issues. For the rapid dissolution of such drugs, it is essential that the polymers used for FDM 3D-printing are water-soluble and the drugs are molecularly or amorphously dispersed in polymers during HME and remain such in the filaments as well as in the tablets [30]. The tablets should also be physically stable during their shelf-lives such that no crystallization of drugs occurs. Therefore, drug-polymer miscibility is critically important for the development of FDM 3D-printed tablets, especially for drugs with poor aqueous solubility. The drug-polymer miscibility will also dictate how much drug can be loaded in the filaments and then in the tablets. However, in most reported studies on FDM 3D-printing in the literature, not much consideration of drug-polymer miscibility is made, and it has been reported that the poorly water-soluble drugs may remain either partially or fully crystalline in the tablets [31]. In one of the few studies where drug-polymer miscibility was determined for the development of FDM 3D-printed tablets, Solanki et al. [19] tested the miscibility of haloperidol with polyvinyl pyrrolidone-vinyl acetate copolymer (Kollidon® VA64), hydroxypropyl methylcellulose (Affinisol® 15cP) and the 1:1 mixture of the two polymers by applying the film casting technique previously developed by Parikh et al. [32]. They observed that haloperidol was miscible with Kollidon® VA64, Affinisol® 15cP and the 1:1 Kollidon-Affinisol mixture to the extents of approximately 20, 10 and 15 percent, respectively. In another study, Wei et al. [33] observed that the miscibility of haloperidol with another polymer, polyvinyl alcohol (PVA), was <10 % w/w, while a different drug, carvedilol, was miscible with PVA up to 20 % w/w. These reports demonstrated that the miscibility of drugs with polymers could be rather limited, and the extent of drug-polymer miscibility may depend on the properties of both drugs and polymers used. It may also be noted that if the drug-polymer miscibility and, thereby, the drug loading is low, the size of printed tablets may become too large for certain doses.

### Need for high processing temperature

The hot melt extrusion (HME) temperature to prepare filaments for FDM 3D-printing should be sufficiently high that the polymers must exist in the molten state above their glass transition temperatures, and their melt viscosities should be within a certain range such that they can be extruded into filaments [19,20,24,34]. Although the presence of the drug may reduce the melt viscosity and thus the extrusion temperature by the plasticization of polymers [34,35], the melt extrusion temperatures in most of the reported studies in the literature are still high. When the filaments are passed through the printer, as mentioned earlier, the temperature within the printer nozzle must be even higher to further soften the filaments such that they can be deposited layer by layer into tablets. By surveying 58 FDM 3D-printed tablets reported in the literature, Cailleaux et al. [26] reported that filaments for 50 of them were melt extruded at temperatures ranging from 100 to 210 °C, where temperatures of 140-180 °C were common. Among these tablets, 47 were printed in the temperature range of 150 to 250 °C, where temperatures around 200°C were the most used. Thus, high temperatures required for the HME of filaments and even higher temperatures needed for 3D printing could be major issues for the thermal stability of drugs and polymers. At such high melt extrusion and printing temperatures, drugs, polymers, or both may be thermally unstable. It is essential that the processing temperatures are lowered to make FDM 3D-printing more widely applicable to drug product development.

### Drug release rate

Lastly, the most difficult challenge in the development of FDM 3D-printed tablets is to ensure rapid drug release from the tablets. The drug-loaded filaments produced by HME are essentially nonporous masses, which are then printed into compact and hard tablets. Therefore, the tablets dissolve in aqueous media by erosion from the surface without any disintegration into particles, which often leads to slow drug release rate. Indeed, Cailleaux et al. [26] reported that out of 56 FDM 3D-printed drug products for which drug release rates were reported in the literature, 43 were slow-release dosage forms, 4 were both sustained and immediate release dosage forms, and 9 were for immediate release. However, as will be discussed in the next section, the rapid drug release rate is critically important for most drugs for optimal therapy [17,18]. Therefore, as mentioned earlier, the primary focus of the present commentary is to assess the status of the development of rapidly dissolving FDM 3D-printed tablets. The significance of rapid drug release in drug therapy will also be discussed, which will then be followed by the discussion of various strategies employed to increase drug release rates from FDM 3D-printed tablets.

## Why rapid drug release?

The therapeutic outcome of drug products and optimal patient benefits depend greatly on how drugs are released from dosage forms and reach the target sites after oral administration and how the drug levels are maintained in the body. Therefore, it is essential that the optimal drug release attributes must be built into dosage forms.

**Figure 2.**
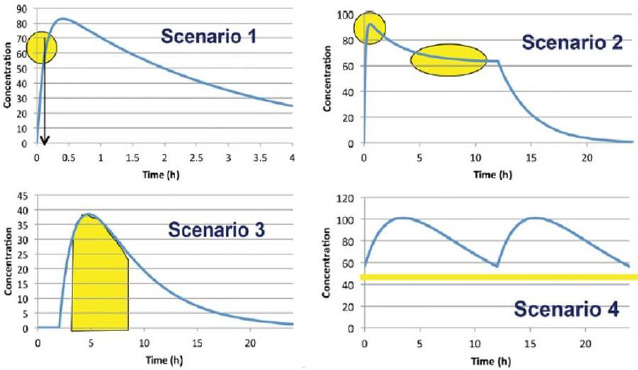
Four scenarios of drug product development according to the Biopharmaceutical Roadmap (BioRAM). Adapted from Selen et al. [17] with permission.

In 2014, an international panel of experts representing the Food and Drug Administration (FDA), academia, and the pharmaceutical industry from the US and Europe, headed by Dr. Arzu Selen from the FDA, developed a Biopharmaceutical Risk Assessment Roadmap (BioRAM) to delineate optimal drug delivery rates and patterns for achieving the desired therapeutic outcomes [18]. The team also described different biopharmaceutics tools that may be applied to identify and address potential challenges to optimize drug products for patient benefits [17]. According to BioRAM, blood levels of different drugs in the body may be classified into four scenarios, as shown in Figure 2, to achieve the desired therapeutic outcomes:

- *Scenario 1* represents drugs where very rapid drug absorption and therapeutic action are needed for such therapies as breakthrough pain, analgesia, acute angina pectoris, insomnia, etc.
- *Scenario 2* represents multiphasic delivery of a single drug or multiple drugs from the same dosage form that has both rapid action and sustained action, and this scenario may include drug products for attention deficit hyperactivity disorder (ADHD), combination histamines, antimigraine drugs, drugs for sleep disorders where the rapid onset must be combined with sustained sleep, and so forth.
- *Scenario 3* represents drug products where the drugs must be rapidly released and absorbed at a predetermined time and then the exposures are maintained, and this scenario may include drug products for the maintenance of blood glucose levels to match the physiological effects of food intake, certain oncology drugs targeted for tumor growth, antihypertensive agents targeted to address circadian relief of blood pressure, etc.
- *Scenario 4* includes drug products where the targeted exposure in the body is maintained by repeated dosing, and the drug must be absorbed rapidly enough that its concentration after each dose reaches the therapeutic level. Many oral antibiotics, antihypertensives, antiepileptics, anti-Alzheimer’s disease drugs, chronic pain medications, antispasmodics, etc., belong to this scenario.

It is evident from the above-mentioned scenarios that drugs must be rapidly released from products that fall under *Scenarios* 1 and 2, and drugs must also be released rapidly under *Scenario 3* after a certain initial lag time. Even when a drug product is developed for prolonged or sustained release, as depicted by *Scenario 4*, there is usually a rapid-release aspect of a drug product so that the therapeutic blood levels are attained rapidly after initial or repeated dosing. Thus, whatever is the scenario of drug product development, a rapid-release attribute must be built into the product. Since most FDM 3D-printed tablets result in slow-release products, further development of the technology to achieve rapid drug release is needed before it can be applied for the development of therapeutically effective drug products according to different BioRAM scenarios.

## Current strategies for the development of rapid-release FDM 3D-printed tablets

Several strategies for the development of rapidly dissolving FDM 3D-printed tablets have been reported in the literature. They include selection of rapidly dissolving polymers with optimum attributes for 3D printing, use of polymer-polymer mixtures, incorporation of additives, modification of internal patterns of tablets and tablet geometry, and supersolubilization of a drug by acid-base interaction.

### Selection of rapidly dissolving polymers

The most convenient approach to increase the dissolution rates of drugs from the FDM 3D-printed tablets could be the use of highly water-soluble and rapidly dissolving polymers. However, as mentioned earlier, there is only a limited number of polymers available that are water-soluble and printable. Polyvinylpyrrolidone (PVP) and related polymers, which are highly water-soluble, may not be used for FDM 3D printing as they produce brittle filaments that are not printable [19-21]. Funk et al. [36] reviewed the literature to determine the effects of polymers on drug release rates from 3D-printed tablets and observed that among various polymers, only Eudragit® E, which is a polymethacrylate polymer, could possibly provide the most rapid drug release rate. The authors observed that, among other polymer types, celluloses and polyvinyl alcohols (PVA) usually provide slow drug release rates from the FDM 3D-printed tablets, and alternative 3D-printing techniques like semisolid extrusion (SSE), selective laser sintering (SLS), digital light processing (DLP), etc., are necessary to increase dissolution rates of drugs from such polymers.

The reported high dissolution rate of drugs from tablets containing Eudragit® E and its impact on drug absorption after oral administration, however, need further scrutiny before it is selected for FDM 3D-printing. Eudragit® E (orally administered grade known as Eudragit® EPO) is a cationic polymer comprised of dimethyl aminoethyl methacrylate, butyl methacrylate and methyl methacrylate at a ratio of 2:1:1 in the polymeric chain [37]. Because of the protonation of the basic amino group in the structure, it is soluble at low acidic pH and insoluble at pH>5. Several investigators reported relatively rapid drug release (<1 h) when the dissolution testing of the FDM 3D-printed tablets containing Eudragit® E was conducted in 0.1 M HCl (pH~1.1), and they did not study the dissolution of the tablets at higher intestinal pH conditions (pH>5), where the polymer is insoluble [38-40]. The dissolution testing was often conducted in 0.1 M HCl, even for the amorphous solid dispersion formulations of drugs in Eudragit® E reported in the literature [41,42]. In a few cases where the dissolution was studied at both lower gastric and higher intestinal pH conditions, dissolution rates and extents of drug release at the higher pH of 6.8 were much lower than that at the lower pH [43,44]. Considering that the average pH of the gastric fluid is around 2 in the fasted state, which may increase to >5 in the fed state [45], the pH-dependent solubility of Eudragit® E may have a major impact on drug release from the FDM 3D-printed tablets after oral administration. The shorter gastric residence time in the fasted state (~30 min) could also be a factor in drug dissolution and absorption since the tablet may pass rapidly through the stomach before all the drugs could be released, and then it may not dissolve in the intestine since the polymer is not soluble at the higher pH.

Careful consideration must also be given to the printability of filaments containing Eudragit® E. Since the polymer has a low glass transition temperature (52 °C), the filaments could be very soft at the relatively high printing temperatures used. In most of the reported studies, high amounts of solid drugs and solid additives were added to the polymer to reduce its fluidity after melting. For example, Yang et al. [46] added 37.5-50 % talc to Eudragit® E-based formulations to make the filaments harder and printable.

### Use of polymer mixtures

Another approach to increase the drug release rate from FDM 3D-printed tablets that has been introduced by Solanki et al. [19] is using mixtures of polymers. In this way, the polymers that are water-soluble but not normally printable could be printed by mixing with other polymers. The authors mixed polyvinylpyrrolidone-vinyl acetate copolymer (Kollidon® VA64) with hydroxypropyl methylcellulose (Affinisol® HME 15 cP) at 1:1 w/w ratio to increase the dissolution rate of a model drug haloperidol as compared to that with cellulose alone. They obtained complete drug release in <1 h at pH 2, and it took ~3 h to completely dissolve the drug at pH 6.8 because of the pH-dependent solubility of the drug used. While Kollidon® VA64 is not printable due to the brittleness of filaments, mixing with Affinisol® HME 15 cP made the formulations printable. This study will be further elaborated in a later section of this article as a case study for a systematic approach to the development of FDM 3D-printed tablets.

In another study, Saydam and Takka [47] used 20 % Kollidon® VA64, 20 % HPMC and 34 % Soluplus® along with different drugs (15 %) and several excipients like Tween 80, Gelucire 44/14, Gelucire 48/16, triacetin, etc., to increase dissolution rate and printability of formulations. Up to 80 % drug release in <1 h was obtained from several formulations for tablets with 50 % infill, while the dissolution from other formulations took longer.

### Incorporation of additives

It has been reported in the literature that the incorporation of certain fillers in FDM 3D-printed tablets could also increase the dissolution rate of drugs from tablets. Okwuosa et al. [48] observed complete release of theophylline or dipyridamole in 30 min from tablets containing 50 % polyvinylpyrrolidone (PVP; MW 40,000 ) as the polymer, 12.5 % triethyl citrate (TEC) as the plasticizer, 27.5 % talc as the filler, along with 10 % drug load. Talc remained solid and fully crystalline, and the drugs were partially crystalline in the tablets. The exact mechanism of how the additives increased drug release rates has not been elucidated in the study. It has been postulated that the matrix structure of tablet influenced the drug release rate. Sadia et al. [49] also observed similar results in the presence of TEC and a different filler, tricalcium phosphate (TCP), where over 80 % release of four model drugs (5-acetyl salicylic acid, captopril, prednisolone, or theophylline) was obtained in <30 min from tablets containing Eudragit® EPO, TEC, TCP, and drug at the ratio of 46.75:3.25:37.5:12.5. The amorphous tricalcium phosphate dispersed in the tablet as separate particles, and the drug was partially crystalline. The exact mechanism for the improvement of dissolution rates by these additives has also not been elucidated. In addition to increasing the drug dissolution rate, the presence of TCP in the formulation helped in the printing process by enhancing the solidification of different printing layers after extrusion. In another study of the effect of additives, Fanous et al. [50] observed that the incorporation of the hydrophilic plasticizer xylitol (13.5 %] and the pore former maltodextrin (9.5 %) in a Eudragit® EPO-based formulation could enhance drug release from the FDM 3D-printed tablets. By using these additives along with the modification of infill density, they developed tablets with complete dissolution in 30 to 60 min.

There are also reports on the increase in drug dissolution rates by the incorporation of polyethylene oxide as the additive [38,43,51,52]. For example, Gultekin et al. [38] used mixtures of Eudragit® EPO and polyethylene oxide (PEO) N80 at 70:30, 60:40 and 50:50 w/w ratios for a low-dose drug (0.25 to 1 mg) and observed complete drug release in a pH 1.2 medium (0.1M HCl) in 20 min. The authors reported that, when extruded at 130 °C, the filaments were flexible, smooth, and extrudable. It is, however, not clear from the paper whether the high ratios of polymer and PEO used (70:30, 60:40 and 50:50 w/w) were miscible with each other or whether they were phase separated, which could be an issue on the physical stability of filaments and tablets. Also, the dissolution testing was conducted at pH 1.2, where the polymer Eudragit® EPO is soluble; it is not known what the dissolution rates of formulations at higher intestinal pH conditions (pH 5.5 to 7.5) would be since the polymer is not soluble at such pH.

Unlike Gultekin et al. [38], the experience of Alhijjaj et al. [43] in using PEO and related additives was quite mixed. They prepared FDM 3D-printed discs containing 10 % felodipine by using 50 % of either Eudragit® EPO or Soluplus as the polymeric matrix and adding 15 % PEO, 15 % Tween 80 and 10 % polyethylene glycol (PEG) 4000 to the formulations. The effects of PEO and the other additives on drug release from Eudragit® EPO- and Soluplus®-based 3D-printed discs were different. While complete drug dissolution was observed from the Eudragit® EPO-based formulation at the gastric pH condition of 1.2 in <30 min, the drug release from the same formulation was much lower at the intestinal pH condition of 6.8 as only ~20 % drug dissolution was observed in 6h, thus showing the effect of pH on drug release. For the Soluplus®-based discs, on the other hand, the drug release rates were extremely low at both pH 1.2 and 6.8 despite the presence of PEO. Moreover, PEO and PEG 400 were not miscible with polymers like Eudragit® EPO and Soluplus® as differential scanning calorimetric (DSC) and powder X-ray diffraction (PXRD) analyses indicated the existence of separate phases in the tablets (discs). Such results indicated that the incorporation of low-melting PEO and other additives might not be the solution to the dissolution issue with FDM 3D-printed tablets.

In a different study, Kollamaram et al. [53] increased the dissolution rate and decreased the printing temperature of the FDM 3D-printed tablets by incorporating 20 % PEG 1500 and 10 % mannitol in a formulation containing 3 % ramipril (drug) and 65 % Kollidon® VA64 or a mixture of Kollidon® VA64 and Kollidon® 12PF. The authors reported almost complete drug release in <30 min when the filaments were extruded at 65-70 °C and the tablets were printed at 90 °C. However, since the glass transition temperature of both the polymers was 109 °C [54], which is much higher than the processing temperatures of filaments and tablets, it is not clear from the report whether the relatively low melting PEG 1500 (m.p.: 43-49 °C) was miscible with Kollidon® VA64 and Kollidon® 12PF at extrusion and printing temperatures used. It could be possible that PEG and polymers were only physically mixed together in the filaments due to the melting of PEG, and it could also be possible that the rapid drug release was observed because of the dispersion of the low concentration drug (3 %) in the higher amount of PEG (20 %) and the polymers did not play any role in the dissolution process.

Attempts were also made to increase dissolution rates of drugs from the FDM 3D-printed tablets by the incorporation of such super-disintegrants as sodium starch glycolate, croscarmellose sodium, and cross-linked polyvinylpyrrolidone (Kollidon® CL) with a mixture of PVA and HPMC used as the polymeric matrix [55]. Although the drug release rate in aqueous media could be increased from 58 % in 5 h to almost complete release in 2 h by this approach, it was not a sufficient increase in dissolution rate for the development of a rapidly dissolving tablet. A faster drug release could not be obtained possibly because when the disintegrants were extruded with the polymers, they lost their swelling properties and disintegration efficiency since the disintegrants were completely surrounded by the polymers preventing any penetration of water to the disintegrants and there was also no porosity within the filaments for swelling of the disintegrants.

Shi et al. [56] observed that the incorporation of release-modifying excipients like poly(vinyl alcohol) (PVA), Soluplus®, polyethylene glycol (PEG) 6000, Eudragit® RLPO/RSPO, HPMC K4M/E10M/K100M, Kollidon® VA64, etc., may also not be the solution to slow drug release from FDM 3D-printed tablets. They used various release modifiers to modulate dissolution rates of ibuprofen from the FDM 3D-printed tablets containing ethyl cellulose as the polymeric matrix. It took over 24 h for the complete release of the drug because of the nature of the polymer and release modifiers used.

The incorporation of additives is a promising and potentially viable approach for increasing the dissolution rates of drugs from the FDM 3D-printed tablets. Indeed, Krueger et al. [57] called the polymer-additive adducts hybrid materials since the additives can impart new properties to the polymers. Resulting in better performance. However, further research is necessary for a better understanding of the interaction between polymers and different additives and its effects on the drug release rate. From the above brief review of the literature, it is apparent that certain additives can plasticize polymers to make them extrudable and printable, and some other additives, especially the solid ones like talc and TCP, may also improve the printability of filaments by making them harder. Care must, however, be taken in selecting additives for such effects since they do not always exert desired effects. In one study, Wei et al. [33] observed that when relatively low melting additives like PEG 3350 or PEG 8000 were mixed with polyvinyl alcohol for plasticization of the polymer during melt extrusion into filaments, the additives melted and oozed out the extruder before exerting any plasticization effects. There could also be phase separation of additives during the stability testing of filaments and tablets. For these reasons, it is essential that the additives, especially the low-melting ones, are miscible with the polymers.

### Modification of tablet pattern and geometry

**Figure 3.**
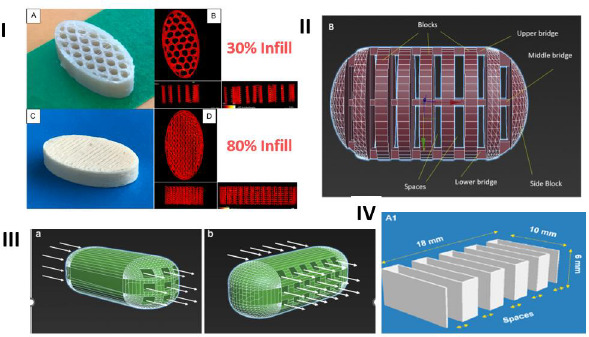
Different methods of increasing surface area by modifying tablet patterns and geometry: I, changing tablet infill; II, geometric modification by connecting blocks of fill materials; III, introduction of perforated channels; and IV, connection of parallel plates in a radiator-like design.

As mentioned earlier, the FDM 3D-printed tablets are hard and non-disintegrating compacts that usually dissolve by erosion. Therefore, one common strategy to increase the dissolution rate of drugs from the tablets is to increase the dissolving surface area of tablets by modifying tablet geometry. Some of these approaches, as shown in Figure 3, are described below:

*Changing tablet infill* - The most common approach to increase the dissolving surface area of the FDM 3D-printed tablet is to decrease the infill of tablets, where, as shown in Fig. 3-I, the infill represents the volume of a tablet taken up by the fill material during the printing of the tablet. The infill of an FDM 3D-printed tablet may vary from less than 10 % to 100 %, indicating that a tablet with, for example, 30 % and 80 % infills will have 30 % and 80 % filled and the remaining 70 % and 20 % will, respectively, be empty spaces or voids within the tablets, while a tablet with 100 % infill will be totally filled and there would not be any empty space inside. Thus, the same weight of tablet with a lower infill will have a higher dissolving surface and, therefore, a higher dissolution rate in the dissolution medium than a tablet with a higher infill [58]. Although the lowering of infill density may increase dissolution rates of drugs from FDM 3D-printed tablets, it may not necessarily lead to a product that may be called rapid or immediate release. In a study of the effect of infill density on the dissolution of a weakly acidic compound, 2-(4-isobutylphenyl) propionic acid, from FDM 3D-printed tablets containing hydroxypropyl methyl acetate succinate (HPMCAS-MG), Thakkar et al. [58] observed that it took ~7h for 80 % drug release at pH 6.8 from tablets with 80 % infill. The drug release rate increased progressively when the fill densities were decreased to 60, 40 and 20 %. However, even at 20 % infill, the drug release rate was relatively slow (80 % drug release in ~2 h). Considering that HPMCAS-MG is soluble at pH 6.8, such release rates are not optimal for the development of rapidly dissolving drug products. In another study from the same laboratory, Zhang et al. [59] obtained complete drug release from ibuprofen-HPMC-MG tablets in 3-4 h from FDM 3D-printed tablets with 25 to 75 % infill density. Such slow-release rates were also observed by Goyanes et al. [60] from FDM 3D-printed tablets of the organic dye fluorescein containing polyvinyl alcohol, a water-soluble polymer, as the matrix. They observed complete release of the dye in aqueous media from tablets with 10 % infill in 6 h, while only about 70 % release was observed at the same period from tablets with 50 and 90 % infills.
*Geometric modification by connecting blocks of fill materials* - In addition to changing the infill density, several other geometrical modifications to increase drug release rates have also been reported in the literature. In one such geometric modification shown in Fig. 3-II, Arafat et al. [61] used a blend of theophylline, hydroxypropyl cellulose and triacetin (50:45:5) for the development of FDM 3D-printed tablets, where they connected 9 blocks of fill materials with 3 bridges within a tablet by leaving gaps ranging from 0 to 1.2 mm between the blocks. The drug release rate from tablets depended on the size of the block, the number of bridges and the spacing between the blocks. It took almost 2 h for the complete dissolution of the drug when the gap in between blocks was 0.2 mm. On the other hand, when the gap was increased to 1 mm or higher, over 80 % theophylline dissolved in 30 min; the higher gap was necessary for the collapse of the bridge and the disintegration of tablet.
*Introduction of perforated channels* - In another approach to increase the dissolution rate of tablets, Sadia et al. [62] introduced perforated channels within the FDM 3D-printed tablets for exposure of the larger surface area to dissolution media (Fig. 3-III). The capsule-shaped tablets (caplets) contained channels of increasing width (0.2, 0.4, 0.6, 0.8 or 1.0 mm) and variable length that were aligned parallel or at a right angle to the long axis of tablet. Over 80 % of drug release in 30 min at pH 1.2 was observed when the channel width was ≥ 0.6 mm. However, the formulation for the tablet was composed of a mixture of hydrochlorothiazide (HCT), Eudragit® E, tricalcium phosphate (TCP) and triethyl citrate (TEC) at the ratio 12.5:46.75:25.37:3.25 w/w. As was discussed earlier, TCP plays a major role in increasing dissolution rates of drugs from 3D-printed tablets, and it is not clear what roles TCP played in increasing dissolution rates of HCT from this formulation. Further, Eudragit® E is soluble under gastric pH conditions (pH<5) but insoluble at higher intestinal pH conditions. Therefore, it is also essential to consider how much improvement in dissolution rate the perforated channels may provide at pH>5 from formulations containing Eudragit® E. Additionally, the dissolution rate improvements from tablets by low infill density and by perforated channels appear to be based on similar principles, and it is essential to determine whether one method would have an advantage over the other.
*Connection of parallel plates* - In yet another geometric modification to increase dissolution rates of tablets, Isreb et al. [63] connected parallel plates of infill in a radiator-like design with inter-plate spacings of 0.5, 1, 1.5, or 2.0 mm (Fig. 3-IV). Filaments used for this study contained 30:35:35 w/w mixtures of theophylline, polyethylene glycol (PEG) 6000, and different grades of polyethylene oxide (PEO) with molecular weights ranging from 100,000 to 900,000, where theophylline was not completely miscible with other components and remained mostly crystalline. A minimum inter-plate spacing of 1 mm was deemed necessary for increasing the drug release rate, where over 80 % of theophylline was released from FDM 3D-printed tablets in <30 min at pH 1.2. The radiator-like design possibly helped in the swelling and disintegration of tablets, whereby crystalline theophylline was liberated in the dissolution medium.

Among other variations of tablet patterns and geometry, Khaled et al. [64] introduced mesh structures within tablets to increase surface area and dissolution rates of 3D-printed paracetamol tablets that appear to be analogous to changing the infill density. Other investigators changed tablet shapes and dimensions to increase the dissolution rates of tablets [65,66]. For example, Goyanes et al. [65] printed tablets with five different geometries, namely, cube, pyramid, cylinder, sphere, and torus, and observed that the drug release rate from the tablets was dependent on the surface area to volume ratio since, as expected, the higher the surface to volume ratio was, so was the dissolution rate. Raje et al. [67] introduced a new tablet geometry where they applied different numbers of outer shells to the FDM 3D-printed tablets to modulate drug release from tablets containing a water-soluble polymer, poly(2-ethyl-tetra-oxazoline) [PETOx], and two different drugs (dextromethorphan hydrobromide and hydrochlorothiazide). The drug release rate increased with the decrease in the number of outer shells. The release rates of dextromethorphan hydrobromide from one-shell and five-shell tablets were, respectively, >80 % and >60 % in 1 h, and because of the lower solubility of hydrochlorothiazide, the release rate was much lower as it took 2 h for 80 % drug release from a one-shell tablet.

Although the modification of tablet geometry could be an attractive approach to increase dissolution rates of FDM 3D-printed tablet, Melocchi et al. [68] reported that computer-aided design (CAD) of such tablets could be very challenging as they involve detailed and complex digital models, and the users may need extensive training and years of experience to master all the functionalities. This is possibly the reason why each geometric design of 3D-printed tablets reported in the literature, except for the infill density, is essentially one of a kind and there is not much follow-up of the technique by other investigators. Hopefully, the technology will grow rapidly enough that the geometric modification will be easy to design and will become more user-friendly. In addition to the complexity of designing tablets, one should keep in mind that the geometric modification may improve the dissolution rate only to a certain extent, and it may not always lead to immediately dissolving or rapidly dissolving tablets, which also depends on types of polymers used and compositions of formulations.

### Application of acid-base supersolubilization principle

**Figure 4.**
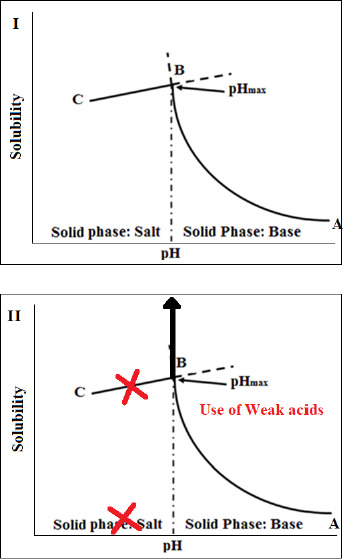
Graphical illustration of acid-base supersolubilization (ABS) principle for a basic drug, where Graph I (top) represents a typical pH versus solubility profile resulting in salt formation at low pH, and Graph 2 (bottom) shows a high increase in solubility (supersolubilization) with lowering of pH when no salt is formed by the addition of a weak acid.

Another promising approach to increase dissolution rates of FDM 3D-printed tablets that has been recently introduced by Patel and Serajuddin [20,21] is the application of acid-based supersolubilization or ABS principle in the development of rapidly dissolving haloperidol-glutaric acid and haloperidol-malic acid tablets, where an otherwise brittle polymer, Kollidon® VA64, was made printable, the loading in the tablet was increased, melt extrusion and printing temperatures were lowered, and the drug release rate was increased. The ABS principle, as described in Figure 4, represents a novel strategy of greatly increasing the aqueous solubility of weakly basic drugs and thereby increasing drug dissolution rates by interaction with weak acids that would not normally form salts with the drugs [69-71]. This principle is based on classical pH-solubility theory [72,73], where, as shown in Fig. 4-I, the aqueous solubility of a basic drug increases according to line A→B when the pH of an aqueous medium is lowered by the addition of acid until pH_max_ or the pH of maximum solubility (Pont B), is reached. A salt is formed below pH_max_, where the drug solubility is dictated by the solubility of its salt (line B→C). However, as shown in Fig. 4-II, if the added acid is not strong enough to lower pH below pH_max_, no salt is formed, and the solubility of the basic drug keeps on increasing, leading to supersolubilization. Singh et al. [70] observed that the aqueous solubility of the model drug, haloperidol, could be increased from the free base solubility of 1 μg/mL to >300 mg/mL when weak acids like malic, tartaric, and citric acids were used to decrease pH. When the highly concentrated solutions were dried, they formed amorphous solids. Parikh et al. [74] demonstrated that such an acid-base interaction leading to the formation of amorphous drugs can also occur in a solid state when mixtures of basic drugs with weak acids are heated together or when the acid-base mixtures were melt extruded along with polymers. In contact with water, the amorphous drugs can exhibit supersolubilization and high dissolution rates. The application of such findings in greatly increasing dissolution rates of basic drugs from the FDM 3D-printed tablets will be illustrated later in Case Study 2. Some recent findings in our laboratory demonstrated that the ABS principle is also applicable to increasing dissolution rates of acidic drugs from FDM 3D-printed tablets by interaction with weak bases.

## Recent progress in development of FDM 3D-printed tablets – Case studies

As evident from the above review of literature, ensuring rapid drug release is a major challenge in the development of successful FDM 3D-printed tablets. Most studies to increase drug release rates from such tablets reported in the literature have been preliminary and exploratory in nature. However, intense efforts towards the development of new strategies for the formulation of rapidly dissolving FDM 3D-printed tablets are continuing. Two emerging strategies that may have major impacts in the development of rapidly dissolving FDM 3D-printed tablets in the future are described below by using case studies.

### Case Study 1: Development of FDM 3D-printed tablets by systematic screening of polymers for rapid drug release

As mentioned earlier, only a very limited number of pharmaceutically acceptable polymers are available for FDM 3D printing. Most polymers used in pharmaceutical dosage forms may not be extrudable into filaments and even those that could be extruded may not be printed since the filaments produced may be either too brittle or too soft. Even when a polymer is both extrudable and printable, it may have other disadvantages like lack of drug-polymer miscibility, slow drug release rate, and the need for high extrusion and printing temperatures where drug, polymer or both may degrade. Therefore, it is essential that polymers and other additives are carefully selected for the successful development of FDM 3D-printed tablets. However, in most of the reported studies, the selection of polymers and other excipients appears to be either arbitrary or by trial and error. To address these issues, Solanki et al. [19] developed a systematic approach for identifying polymers or polymeric blends for the development of rapidly dissolving FDM 3D-printed tablets. Later, Aho et al. [75] also developed a roadmap for the development of FDM 3D-printed tablets based on material properties and the processing behavior of drug-excipient combinations, but their focus was on the printing of tablets rather than drug release rate.

**Figure 5.**
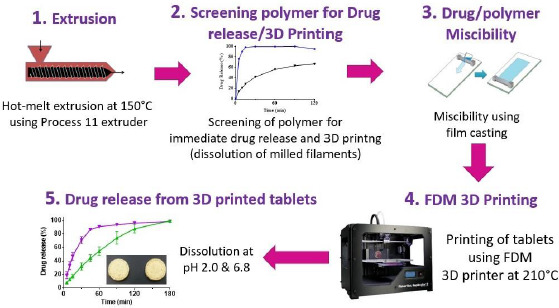
Systematic approach for the identification of polymers for the development of rapidly dissolving FDM 3D-printed tablets.

The strategy developed by Solanki et al. {19] for the identification of polymers for rapid drug release, as shown schematically in Figure 5, involved the following steps:

1. Hot melt extrusion of haloperidol with different pharmaceutical grade polymers to determine whether filaments would be formed and whether the drug is molecularly or amorphously dispersed in the polymers to form amorphous solid dispersion (ASD),
2. Evaluate drug release from crushed extrudates for a preliminary assessment of whether the polymer would provide immediate drug release and, if the drug dissolves rapidly from the extrudates, whether the filaments would be printable,
3. Determine the miscibility of the drug with polymer or mixtures of polymers to identify the maximum drug load that may be used in the filaments identified in Step 2 above,
4. Print tablets by FDM using drug-loaded filaments with selected polymers or polymer blends using optimum drug loads determined by drug-polymer miscibility testing in Step 3 above.
5. Evaluate drug release from FDM 3D-printed tablets to determine whether they meet the target drug release.

Solanki et al. [19] screened four commonly used polymers, namely, Kollidon VA64 (vinylpyrrolidone-vinyl acetate copolymer), Kollicoat® IR (polyvinyl alcohol-polyvinyl glycol graft copolymer), Affinisol HPMC HME 15cP (hydoxypropyl methylcellulose HME), and Aquoat AS-MG (hydroxypropyl methylcellulose acetate succinate) for their suitability in the development of rapidly dissolving FDM 3D-printed tablets by using a model basic drug, haloperidol (pK_a_~8), with pH-dependent solubility (1-4 mg/mL at pH 1-3; 2-3 μg/mL at pH~7). In *Step 1* of the process, they observed that drug formulations with all four polymers were extrudable when they were extruded at a common temperature of 150 °C. In *Step 2*, the dissolution testing of crushed filaments was conducted at pH 2 and 6.8, and the printability of whole (uncrushed) filaments was studied by printing actual tablets. It was observed that the crushed filaments containing Kollidon VA64 only provided the most rapid drug release rate, but the filaments were not printable. For this reason, 1:1-mixtures of Kollidon® VA64 with Affinisol as well as HPMCAS MG were also tested for dissolution and printability, and the results are presented in Table 2. Although slower than that with Kollidon® VA64 alone, the drug-loaded filaments containing the 1:1-mixtures of Kollidon® VA64 with Affinisol was still quite rapid. Filaments containing Kollicoat® IR or 1:1-mixtures of Kollidon® VA64 with HPMCAS MG exhibited delayed drug release rate. Based on the results presented in Table 2, filaments containing 1:1 Kollidon VA64 and Affinisol were selected for further development. The miscibility of haloperidol with Kollidon VA64, Affinisol and their mixtures was then studied in *Step 3* using the film casting technique previously developed by Parikh et al. [32]. The miscibility of the drug was 20 % in Kollidon VA64, 10 % in Affinisol and 15 % in the Kollidon VA64-Affinisol mixture. Based on these results, the formulation composition selected for 3D printing was 10 % haloperidol, 45 % Kollidon VA64 and 45 % Affinisol.

**Figure 6.**
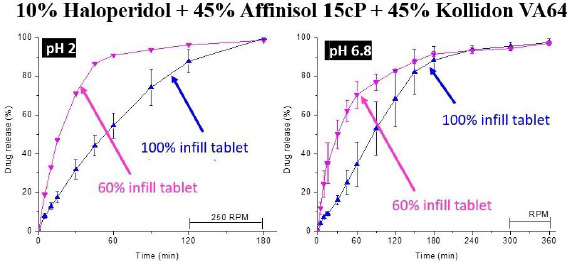
Dissolution of haloperidol from FDM 3D-printed tablets containing 10 % drug load in 1:1 Kollidon® VA64-Affinisol® 15 cP mixtures and having 100 % and 60 % infill densities at pH 2 and 6.8.

The selected formulation was then printed into tablets at 210℃ in *Step 4* of the process. Two infill densities, 100 % and 60 %, were used to determine whether there would be any effect of infill density on drug release. As shown in Figure 6, the dissolution testing of the FDM 3D-printed tablets in *Step 5* gave ~80 % of drug release from tablets with 60 % infill in 45 and 120 min at pH 1.2 and 6.8, respectively. From tablets with 100 % infill, over 80 % of the drug was released at pH 1.2 and 6.8 in 2 and 3 h, respectively.

Although the dissolution rate of haloperidol obtained by Solanki et al. [19] did not meet the FDA guideline of 85 % and higher drug release in 60 min for immediate drug release [76], the rate was still rapid enough for complete drug release in 2-3 h, indicating the potential for relatively rapid and complete drug release during the gastrointestinal residence time [77]. Thus, such a systematic approach demonstrates how 3D-printed tablets with relatively rapid drug release may be identified. If the drug release rate of the FDM 3D-printed tablet thus obtained does not meet the target profile, modifications of the formulation may be made to further increase the drug release rate. Later studies in our laboratory showed that the formulation was still extrudable into filaments if the amount of cellulose was reduced from 1:1 w/w Kollidon® VA64 to Affinisol® 15cP ratio to 3:1 ratio of the same, and decreasing the amount of cellulose in the formulation resulted in a higher drug release rate from the tablets produced.

Figure 5 also identifies several issues that are common in the 3D printing process. The relatively high temperature (150 °C) used for melt extrusion of filaments and even higher temperature (210 °C) necessary for 3D printing of filaments into tablets could pose issues if the drug is thermolabile. There could also be issues with drug loading in tablets depending on the drug-polymer miscibility. Although the miscibility may differ from drug to drug and polymer to polymer, it is essential that drug loading in tablets should be based on a thorough evaluation of the drug-polymer miscibility. Thus, the systematic identification of such issues during the development program guides the formulators towards evaluating and possibly resolving them.

### Case Study 2: Development of FDM 3D-printed tablets with rapid drug release by acid-base interactions

This case study represents a recent advance in FDM 3D-printed technology that not only provides rapid drug release from the tablets, but, at the same time, also resolves two other major issues faced in the development of FDM 3D-printed tablets, namely, low drug-polymer miscibility and the need for high processing temperature. This case study is based on the acid-base supersolubilization (ABS) principle described earlier.

**Figure 7.**
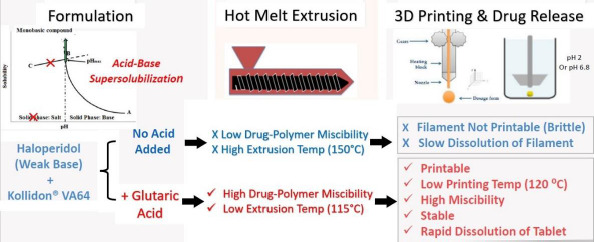
Effects acid-base interaction between glutaric acid and haloperidol on melt extrusion and printing temperatures, printability, and drug-polymer miscibility of FDM 3D printed tablets.

The application of the ABS principle in increasing release rates of drugs from FDM 3D-printed tablets is shown schematically in Figure 7 based on the work of Patel and Serajuddin [20], where the formulation contained haloperidol as the model basic drug, glutaric acid as the super-solubilizing agent, and Kollidon® VA64 as the polymer. In the presence of glutaric acid, the aqueous solubility of haloperidol increased to >300 mg/mL, which indicated that an interaction between haloperidol and glutaric acid occurred, leading to the supersolubilization of the drug. Such an interaction between haloperidol and glutaric acid was also observed in the solid state, which reduced the melt extrusion temperature of the formulation from 150 °C to 115 °C since the haloperidol-glutaric mixture converted to a semisolid mass during melt extrusion that plasticized the polymer. While the haloperidol-Kollidon® VA64 mixture was not printable, the plasticizing effect of the acid-base mixture when glutaric acid was added to the formulation also made the filaments printable and lowered the printing temperature to as low as 120 °C as compared to ~200 °C required in the absence of glutaric acid. The drug was also highly miscible with the formulation to as high as 50 % w/w.

**Figure 8.**
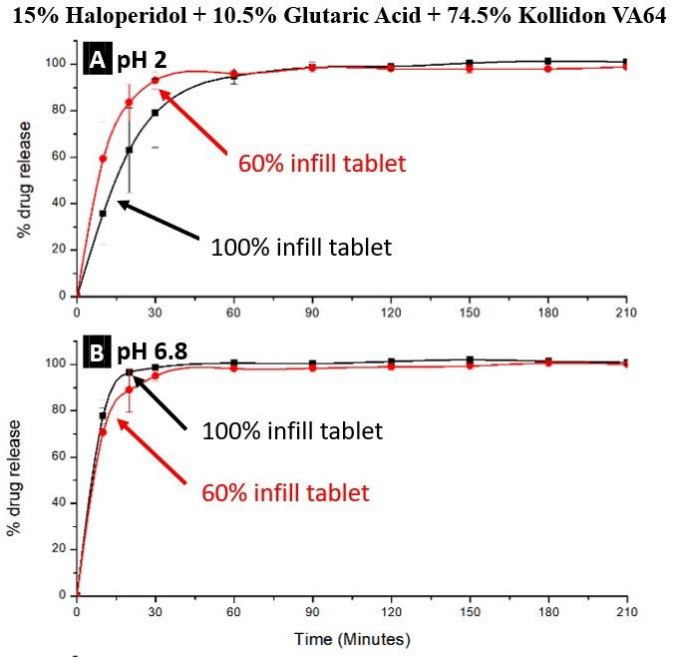
Dissolution of haloperidol from FDM 3D-printed tablets containing 15 % drug load from a formulation containing 1:2 molar ratio of drug to glutaric acid and having 100 % and 60 % infill densities at pH 2 and 6.8. Kol, Hal and GA represent Kollidon® VA64, haloperidol, and glutaric acid.

The drug release rates from tablets containing glutaric acid were rapid. As shown in Fig. 8, almost all the drug dissolved at both pH 2 and 6.8 in 30 min, and because of the high dissolution rate of the drug from such a formulation, there was also no significant effect of the infill density.

Similar results were also obtained for FDM 3D-printed tablets containing haloperidol-malic acid mixtures [21], where the Kollidon® VA64-haloperidol-malic acid ternary mixtures could be extruded into filaments at 120 °C and printed into tablets at 125 and 100 °C, respectively, at 15 % and 30 % drug loads. As high as 50 % w/w drug load could be achieved due to the high miscibility of the drug with the formulation; however, the filaments were too soft with 40 and 50 % w/w drug loads, and, therefore, formulations with only 15 and 30 % w/w drug loads were printed. Over 80 % drug release could be achieved from 100% infill tablets in <30 min at pH 1.2 and 6.8.

## Summary and conclusions

Although there has been a great interest and much research in recent years in the FDM 3D-printing technology for the development of tablets for personalized medicines, it faces many challenges, such as limited availability of suitable polymers, low drug-polymer miscibility, the need for high processing temperatures, and the slow drug release rate that impede the development of clinically efficacious and commercially viable FDM 3D printed tablets. These challenges are discussed in this article with a special focus on the drug release rate. The FDM 3D printing produces compact tablets that dissolve slowly by erosion, while rapid drug release is important for the clinical efficacy of most drug candidates. Such strategies as using rapidly dissolving polymers or polymer-polymer mixtures, incorporation of additives, modification of tablet pattern and geometry, and supersolubilization by acid-base interaction to increase drug dissolution rates are reviewed and the pros and cons of these strategies are discussed. Many polymers, such as PVPs, Kollidon® VA64, etc., are highly water-soluble and could potentially increase dissolution rates of drugs from the FDM 3D-printed tablets, but their filaments are too brittle and cannot be printed. On the other hand, cellulosic filaments are printable, but the tablets produced are slowly dissolving. Mixing cellulose with Kollidon® VA64 is a promising way of increasing dissolution rates of the formulations as the mixtures are printable and, at the same time, they can improve dissolution rates over that with celluloses alone. A polymer like Eudragit® EPO can also produce rapidly dissolving tablets; however, since it dissolves only at pH<5, the dissolution of tablets under intestinal pH conditions at pH>5 could be slow and incomplete. The printability of Eudragit® EPO could also be a concern since it has a relatively low glass transition temperature (~50 °C) and its filaments may require solid additives to make them harder for printing. The incorporation of additives can also increase the dissolution rates of drugs and improve the printability of tablets. However, the rationale for the use of different additives has not been fully elucidated in the literature, and the selection of appropriate additives will require careful consideration and further research. Additionally, there could be phase separation and potential physical instability of filaments and tablets if the additives are not miscible with polymers. Among different methods of modifying tablet patterns and geometry, changing infill density is the most used, and further research and technological progress are necessary for the implementation of other methods. There are, however, limitations to what extent the dissolution rate may be improved by this method. Two emerging strategies for the development of rapidly dissolving FDM 3D-printed are elaborated by using case studies, where one provides a systematic approach for screening polymers for rapidly dissolving tablets, and the other involves the acid-base supersolubilization (ABS) principle that not only increases solubility and dissolution rates of drugs but also increases drug-polymer miscibility and decreases processing temperatures for tablets. Although very promising, most of the strategies applied to the development of FDM 3D-printed tablets are rather at their early stages of development, and further research is needed for the implementation of these strategies into the development of marketed drug products. Many of the gaps in the current state of the FDM 3D-printing technology have been presented in this article, and a few strategies to fill those gaps have been highlighted. Hopefully, they will provide guidance for the future development of the technology.

## Figures and Tables

**Table 1.** The results of the screening of different polymers and polymer-polymer mixtures with 10 % w/w drug loads for drug release from crushed filaments at pH 2 and 6.8 and printability of whole filaments.

Polymer	Printability	Drug release from milled filaments
10 % Haloperidol + 90 % Kollidon VA 64	Breaks into the drive gear; not printable	Immediate
10 % Haloperidol + 90 % Kollicoat IR	Printable	Retarded
10 % Haloperidol + 90 % Affinisol 15 cP	Printable	Retarded
10 % Haloperidol + 45 % HPMCAS MG + 45 % Kollidon VA 64	Printable	Retarded
**10 % Haloperidol + 45 % Kollidon VA 64 + 45 % Affinisol 15 cP**	**Printable**	**Immediate**

## References

[1] Awad A. Goyanes A. Basit A. Zidan A. Xu C. Li D. Narayan R. Chen R.K.. A Review of State-of-The-Art on Enabling Additive Manufacturing Processes for Precision Medicine. Journal of Manufacturing Science and Engineering 145 (2023) 010802. https://doi.org/10.1115/1.4056199 10.1115/1.4056199

[2] Wong K.V. Hernandez A.. A review of additive manufacturing. International scholarly research notices 2012 (2012) 208760. https://doi.org/10.5402/2012/208760 10.5402/2012/208760

[3] Spritam FDA approval history. https://www.drugs.com/history/spritam.html (last accessed: November 12, 2022)

[4] Melocchi A. Uboldi M. Cerea M. Foppoli A. Maroni A. Moutaharrik S. Palugan L. Zema L. Gazzaniga A.. A graphical review on the escalation of fused deposition modeling (FDM) 3D printing in the pharmaceutical field. Journal of Pharmaceutical Sciences 109(10) (2020) 2943-2957. https://doi.org/10.1016/j.xphs.2020.07.011 10.1016/j.xphs.2020.07.011 32679215

[5] Wang J. Zhang Y. Aghda N.H. Pillai A.R. Thakkar R. Nokhodchi A. Maniruzzaman M.. Emerging 3D printing technologies for drug delivery devices: Current status and future perspective. Advanced Drug Delivery Reviews 174 (2021) 294-316. https://doi.org/10.1016/j.addr.2021.04.019 10.1016/j.addr.2021.04.019 33895212

[6] Jamróz W. Szafraniec J. Kurek M. Jachowicz R.. 3D printing in pharmaceutical and medical applications–recent achievements and challenges. Pharmaceutical research 35(9), (2018) 1-22. https://doi.org/10.1007/s11095-018-2454-x 10.1007/s11095-018-2454-x PMC6061505 29998405

[7] Parulski C. Jennotte O. Lechanteur A. Evrard B.. Challenges of fused deposition modeling 3D printing in pharmaceutical applications: Where are we now? Advanced drug delivery reviews. 175 (2021) 113810. https://doi.org/10.1016/j.addr.2021.05.020 10.1016/j.addr.2021.05.020 34029646

[8] Ragelle H. Rahimian S. Guzzi E.A. Westenskow P.D. Tibbitt M.W. Schwach G. Langer R.. Additive manufacturing in drug delivery: Innovative drug product design and opportunities for industrial application. Advanced Drug Delivery Reviews 178 (2021) 113990. https://doi.org/10.1016/j.addr.2021.113990 10.1016/j.addr.2021.113990 34600963

[9] Reddy R.D.P. Sharma V.. Additive manufacturing in drug delivery applications: A review. International Journal of Pharmaceutics 589 (2020) 119820. https://doi.org/10.1016/j.ijpharm.2020.119820 10.1016/j.ijpharm.2020.119820 32891718

[10] Seoane-Viaño I. Trenfield S.J. Basit A.W. Goyanes A.. Translating 3D printed pharmaceuticals: From hype to real-world clinical applications. Advanced Drug Delivery Reviews 174 (2021) 553-575. https://doi.org/10.1016/j.addr.2021.05.003 10.1016/j.addr.2021.05.003 33965461

[11] Trenfield S.J. Awad A. Goyanes A. Gaisford S. Basit A.W.. 3D printing pharmaceuticals: drug development to frontline care. Trends in pharmacological sciences 39(5) (2018) 440-451. https://doi.org/10.1016/j.tips.2018.02.006 10.1016/j.tips.2018.02.006 29534837

[12] Zema L. Melocchi A. Maroni A. Gazzaniga A.. Three-dimensional printing of medicinal products and the challenge of personalized therapy. Journal of pharmaceutical sciences 106(7) (2017) 1697-1705. https://doi.org/10.1016/j.xphs.2017.03.021 10.1016/j.xphs.2017.03.021 28347731

[13] Bandari S. Nyavanandi D. Dumpa N. Repka M.A.. Coupling hot melt extrusion and fused deposition modeling: Critical properties for successful performance. Advanced Drug Delivery Reviews 172 (2021) 52-63. https://doi.org/10.1016/j.addr.2021.02.006 10.1016/j.addr.2021.02.006 33571550 PMC8107127

[14] Tan D.K. Maniruzzaman M. Nokhodchi A.. Advanced pharmaceutical applications of hot-melt extrusion coupled with fused deposition modelling (FDM) 3D printing for personalised drug delivery. Pharmaceutics 10(4) (2018) 203. https://doi.org/10.3390/pharmaceutics10040203 10.3390/pharmaceutics10040203 30356002 PMC6321644

[15] Khaled S.A. Burley J.C. Alexander M.R. Yang J. Roberts C.J.. 3D printing of five-in-one dose combination polypill with defined immediate and sustained release profiles. Journal of controlled release 217 (2015) 308-314. https://doi.org/10.1016/j.jconrel.2015.09.028 10.1016/j.jconrel.2015.09.028 26390808

[16] Khaled S.A. Burley J.C. Alexander M.R. Yang J. Roberts C.J.. 3D printing of tablets containing multiple drugs with defined release profiles. International journal of pharmaceutics 494(2) (2015) 643-650. https://doi.org/10.1016/j.ijpharm.2015.07.067 10.1016/j.ijpharm.2015.07.067 26235921

[17] Dickinson P.A. Kesisoglou F. Flanagan T. Martinez M.N. Mistry H.B. Crison J.R. Polli J.E. Cruañes M.T. Serajuddin A.T. Müllertz A.. Optimizing clinical drug product performance: applying biopharmaceutics risk assessment roadmap (BioRAM) and the BioRAM scoring grid. Journal of Pharmaceutical Sciences 105 (2016) 3243-3255. https://doi.org/10.1016/j.xphs.2016.07.024 10.1016/j.xphs.2016.07.024 27659159

[18] Selen A. Dickinson P.A. Müllertz A. Crison J.R. Mistry H.B. Cruañes M.T. Martinez M.N. Lennernäs H. Wigal T.L. Swinney D.C.. The biopharmaceutics risk assessment roadmap for optimizing clinical drug product performance. Journal of Pharmaceutical Sciences 103 (2014) 3377-3397. https://doi.org/10.1002/jps.24162 10.1002/jps.24162 25256402

[19] Solanki N.G. Tahsin M. Shah A.V. Serajuddin A.T.. Formulation of 3D printed tablet for rapid drug release by fused deposition modeling: screening polymers for drug release, drug-polymer miscibility and printability. Journal of Pharmaceutical Sciences 107 (2018) 390-401. https://doi.org/10.1016/j.xphs.2017.10.021 10.1016/j.xphs.2017.10.021 29066279

[20] Patel N.G. Serajuddin A.T.. Development of FDM 3D-printed tablets with rapid drug release, high drug-polymer miscibility and reduced printing temperature by applying the acid-base supersolubilization (ABS) principle. International Journal of Pharmaceutics 600 (2021) 120524. https://doi.org/10.1016/j.ijpharm.2021.120524 10.1016/j.ijpharm.2021.120524 33775724

[21] Patel N.G. Serajuddin A.T.. Improving drug release rate, drug-polymer miscibility, printability, and processability of FDM 3D-printed tablets by weak acid-base interaction. International Journal of Pharmaceutics (2023), in press 10.1016/j.ijpharm.2022.122542 36566823

[22] Ilyés K. Kovács N.K. Balogh A. Borbás E. Farkas B. Casian T. Marosi G. Tomuţă I. Nagy Z.K.. The applicability of pharmaceutical polymeric blends for the fused deposition modelling (FDM) 3D technique: Material considerations–printability–process modulation, with consecutive effects on in vitro release, stability and degradation. European Journal of Pharmaceutical Sciences 129 (2019) 110-123. https://doi.org/10.1016/j.ejps.2018.12.019 10.1016/j.ejps.2018.12.019 30610954

[23] Govender R. Kissi E.O. Larsson A. Tho I.. Polymers in pharmaceutical additive manufacturing: A balancing act between printability and product performance. Advanced Drug Delivery Reviews 177 (2021) 113923. https://doi.org/10.1016/j.addr.2021.113923 10.1016/j.addr.2021.113923 34390775

[24] Nasereddin J.M. Wellner N. Alhijjaj M. Belton P. Qi S.. Development of a simple mechanical screening method for predicting the feedability of a pharmaceutical FDM 3D printing filament. Pharmaceutical research 35 (2018) 1-13. https://doi.org/10.1007/s11095-018-2432-3 10.1007/s11095-018-2432-3 PMC5982458 29855818

[25] Borandeh S. van Bochove B. Teotia A. Seppälä J.. Polymeric drug delivery systems by additive manufacturing. Advanced Drug Delivery Reviews 173 (2021) 349-373. https://doi.org/10.1016/j.addr.2021.03.022 10.1016/j.addr.2021.03.022 33831477

[26] Cailleaux S. Sanchez-Ballester N.M. Gueche Y.A. Bataille B. Soulairol I.. Fused Deposition Modeling (FDM), the new asset for the production of tailored medicines. Journal of controlled release 330 (2021) 821-841. https://doi.org/10.1016/j.jconrel.2020.10.056 10.1016/j.jconrel.2020.10.056 33130069

[27] Henry S. Samaro A. Marchesini F.H. Shaqour B. Macedo J. Vanhoorne V. Vervaet C.. Extrusion-based 3D printing of oral solid dosage forms: Material requirements and equipment dependencies. International Journal of Pharmaceutics 598 (2021) 120361. https://doi.org/10.1016/j.ijpharm.2021.120361 10.1016/j.ijpharm.2021.120361 33571622

[28] Liu R., Water-Insoluble Drug Formulation, 3rd Edition ed., Boca Raton, 2018. https:/doi.org/10.1201/9781315120492 10.1201/9781315120492.

[29] Pereira G.G. Figueiredo S. Fernandes A.I. Pinto J.F.. Polymer selection for hot-melt extrusion coupled to fused deposition modelling in pharmaceutics. Pharmaceutics 12 (2020) 795. https://doi.org/10.3390/pharmaceutics12090795 10.3390/pharmaceutics12090795 32842703 PMC7558966

[30] Khalid G.M. Billa N.. Solid Dispersion Formulations by FDM 3D Printing—A Review. Pharmaceutics 14 (2022) 690. https://doi.org/10.3390/pharmaceutics14040690 10.3390/pharmaceutics14040690 35456524 PMC9032529

[31] Pinho L.A. Lima A.L. Sa-Barreto L.L. Gratieri T. Gelfuso G.M. Marreto R.N. Cunha-Filho M.. Preformulation studies to guide the production of medicines by fused deposition modeling 3D printing. AAPS PharmSciTech 22 (2021) 1-12. https://doi.org/10.1016/j.jpha.2021.09.010 10.1016/j.jpha.2021.09.010 34729662

[32] Parikh T. Gupta S.S. Meena A.K. Vitez I. Mahajan N. Serajuddin A.T.. Application of film-casting technique to investigate drug–polymer miscibility in solid dispersion and hot-melt extrudate. Journal of Pharmaceutical Sciences 104 (2015) 2142-2152. https://doi.org/10.1002/jps.24446 10.1002/jps.24446 25917333

[33] Wei C. Solanki N.G. Vasoya J.M. Shah A.V. Serajuddin A.T.. Development of 3D printed tablets by fused deposition modeling using polyvinyl alcohol as polymeric matrix for rapid drug release. Journal of Pharmaceutical Sciences 109 (2020) 1558-1572. https://doi.org/10.1016/j.xphs.2020.01.015 10.1016/j.xphs.2020.01.015 32004538

[34] Solanki N. Gupta S.S. Serajuddin A.T.. Rheological analysis of itraconazole-polymer mixtures to determine optimal melt extrusion temperature for development of amorphous solid dispersion. European Journal of Pharmaceutical Sciences 111 (2018) 482-491. https://doi.org/10.1016/j.ejps.2017.10.034 10.1016/j.ejps.2017.10.034 29080855

[35] Gupta S.S. Parikh T. Meena A.K. Mahajan N. Vitez I. Serajuddin A.T.. Effect of carbamazepine on viscoelastic properties and hot melt extrudability of Soluplus®. International Journal of Pharmaceutics 478 (2015) 232-239. https://doi.org/10.1016/j.ijpharm.2014.11.025 10.1016/j.ijpharm.2014.11.025 25448585

[36] Funk N.L. Fantaus S. Beck R.C.R.. Immediate release 3D printed oral dosage forms: how different polymers have been explored to reach suitable drug release behaviour. International Journal of Pharmaceutics (2022) 122066. https://doi.org/10.1016/j.ijpharm.2022.122066 10.1016/j.ijpharm.2022.122066 35926751

[37] Thakral S. Thakral N.K. Majumdar D.K.. Eudragit: a technology evaluation. Expert Opin Drug Deliv 10 (2013) 131-149. https://doi.org/10.1517/17425247.2013.736962 10.1517/17425247.2013.736962. 23102011

[38] Gültekin H.E. Tort S. Acartürk F.. An effective technology for the development of immediate release solid dosage forms containing low-dose drug: fused deposition modeling 3D printing. Pharmaceutical research 36 (2019) 1-13. https://doi.org/10.1007/s11095-019-2655-y 10.1007/s11095-019-2655-y 31250313

[39] Pietrzak K. Isreb A. Alhnan M.A.. A flexible-dose dispenser for immediate and extended release 3D printed tablets. European journal of pharmaceutics biopharmaceutics 96 (2015) 380-387. https://doi.org/10.1016/j.ejpb.2015.07.027 10.1016/j.ejpb.2015.07.027 26277660

[40] Than Y.M. Titapiwatanakun V.. Tailoring immediate release FDM 3D printed tablets using a quality by design (QbD) approach. International Journal of Pharmaceutics 599 (2021) 120402. https://doi.org/10.1016/j.ijpharm.2021.120402 10.1016/j.ijpharm.2021.120402 33640426

[41] Lin X. Su L. Li N. Hu Y. Tang G. Liu L. Li H. Yang Z.. Understanding the mechanism of dissolution enhancement for poorly water-soluble drugs by solid dispersions containing Eudragit® E PO. Journal of Drug Delivery Science Technology 48 (2018) 328-337. https://doi.org/10.1016/j.jddst.2018.10.008 10.1016/j.jddst.2018.10.008

[42] Liu H. Wang P. Zhang X. Shen F. Gogos C.G.. Effects of extrusion process parameters on the dissolution behavior of indomethacin in Eudragit® E PO solid dispersions. International Journal of Pharmaceutics 383 (2010) 161-169. https://doi.org/10.1016/j.ijpharm.2009.09.003 10.1016/j.ijpharm.2009.09.003 19748557

[43] Alhijjaj M. Belton P. Qi S.. An investigation into the use of polymer blends to improve the printability of and regulate drug release from pharmaceutical solid dispersions prepared via fused deposition modeling (FDM) 3D printing. European journal of pharmaceutics biopharmaceutics 108 (2016) 111-125. https://doi.org/10.1016/j.ejpb.2016.08.016 10.1016/j.ejpb.2016.08.016 27594210

[44] Wang X. Zhu Y. Zhao X. Zhang S. Cao M. Wang X. Li W.. Development and characterization of an amorphous Curcumin-Eudragit® E100 Solid Dispersions with improved solubility, stability, and pharmacokinetic properties. Pharmaceutical Development Technology (2022) 1-16. https://doi.org/10.1080/10837450.2022.2141778 10.1080/10837450.2022.2141778 36305800

[45] Washington N. Washington C. Wilson C., Physiological pharmaceutics: barriers to drug absorption, CRC Press, 2000, 0429204590.

[46] Yang Y. Wang H. Xu X. Yang G.. Strategies and mechanisms to improve the printability of pharmaceutical polymers Eudragit® EPO and Soluplus®. International Journal of Pharmaceutics 599 (2021) 120410. https://doi.org/10.1016/j.ijpharm.2021.120410 10.1016/j.ijpharm.2021.120410 33639229

[47] Saydam M. Takka S.. Improving the dissolution of a water-insoluble orphan drug through a fused deposition modelling 3-Dimensional printing technology approach. European Journal of Pharmaceutical Sciences 152 (2020) 105426. https://doi.org/10.1016/j.ejps.2020.105426 10.1016/j.ejps.2020.105426 32544421

[48] Okwuosa T.C. Stefaniak D. Arafat B. Isreb A. Wan K.-W. Alhnan M.A.. A lower temperature FDM 3D printing for the manufacture of patient-specific immediate release tablets. Pharmaceutical research 33 (2016) 2704-2712. https://doi.org/10.1007/s11095-016-1995-0 10.1007/s11095-016-1995-0 27506424

[49] Sadia M. Sośnicka A. Arafat B. Isreb A. Ahmed W. Kelarakis A. Alhnan M.A.. Adaptation of pharmaceutical excipients to FDM 3D printing for the fabrication of patient-tailored immediate release tablets. International Journal of Pharmaceutics 513 (2016) 659-668. https://doi.org/10.1016/j.ijpharm.2016.09.050 10.1016/j.ijpharm.2016.09.050 27640246

[50] Fanous M. Bitar M. Gold S. Sobczuk A. Hirsch S. Ogorka J. Imanidis G.. Development of immediate release 3D-printed dosage forms for a poorly water-soluble drug by fused deposition modeling: Study of morphology, solid state and dissolution. International Journal of Pharmaceutics 599 (2021) 120417. https://doi.org/10.1016/j.ijpharm.2021.120417 10.1016/j.ijpharm.2021.120417 33647418

[51] Gültekin H.E. Tort S. Acartürk F.. Fabrication of three dimensional printed tablets in flexible doses: A comprehensive study from design to evaluation. Journal of Drug Delivery Science Technology 74 (2022) 103538. https://doi.org/10.1016/j.jddst.2022.103538 10.1016/j.jddst.2022.103538

[52] Öblom H. Zhang J. Pimparade M. Speer I. Preis M. Repka M. Sandler N.. 3D-printed isoniazid tablets for the treatment and prevention of tuberculosis—personalized dosing and drug release. AAPS PharmSciTech 20 (2019) 1-13. https://doi.org/10.1208/s12249-018-1233-7 10.1208/s12249-018-1233-7 PMC6373414 30617660

[53] Kollamaram G. Croker D.M. Walker G.M. Goyanes A. Basit A.W. Gaisford S.. Low temperature fused deposition modeling (FDM) 3D printing of thermolabile drugs. International Journal of Pharmaceutics 545 (2018) 144-152. https://doi.org/10.1016/j.ijpharm.2018.04.055 10.1016/j.ijpharm.2018.04.055 29705104

[54] Patel N.G. Serajuddin A.T.. Moisture sorption by polymeric excipients commonly used in amorphous solid dispersion and its effect on glass transition temperature: I. Polyvinylpyrrolidone and related copolymers. International Journal of Pharmaceutics 616 (2022) 121532. https://doi.org/10.1016/j.ijpharm.2022.121532 10.1016/j.ijpharm.2022.121532 35121046

[55] Đuranović M. Madžarević M. Ivković B. Ibrić S. Cvijić S.. The evaluation of the effect of different superdisintegrants on the drug release from FDM 3D printed tablets through different applied strategies: In vitro-in silico assessment. International Journal of Pharmaceutics 610 (2021) 121194. https://doi.org/10.1016/j.ijpharm.2021.121194 10.1016/j.ijpharm.2021.121194 34728321

[56] Shi K. Salvage J.P. Maniruzzaman M. Nokhodchi A.. Role of release modifiers to modulate drug release from fused deposition modelling (FDM) 3D printed tablets. International Journal of Pharmaceutics 597 (2021) 120315. https://doi.org/10.1016/j.ijpharm.2021.120315 10.1016/j.ijpharm.2021.120315 33540000

[57] Krueger L. Miles J.A. Popat A.. 3D printing hybrid materials using fused deposition modelling for solid oral dosage forms. Journal of controlled release 351 (2022) 444-455. https://doi.org/10.1016/j.jconrel.2022.09.032 10.1016/j.jconrel.2022.09.032 36184971

[58] Thakkar R. Pillai A.R. Zhang J. Zhang Y. Kulkarni V. Maniruzzaman M.. Novel on-demand 3-dimensional (3-D) printed tablets using fill density as an effective release-controlling tool. Polymers 12 (2020) 1872. https://doi.org/10.3390/polym12091872 10.3390/polym12091872 32825229 PMC7564432

[59] Zhang J. Thakkar R. Zhang Y. Maniruzzaman M.. Structure-function correlation and personalized 3D printed tablets using a quality by design (QbD) approach. International Journal of Pharmaceutics 590 (2020) 119945. https://doi.org/10.1016/j.ijpharm.2020.119945 10.1016/j.ijpharm.2020.119945 33027633

[60] Goyanes A. Buanz A.B. Basit A.W. Gaisford S.. Fused-filament 3D printing (3DP) for fabrication of tablets. International Journal of Pharmaceutics 476 (2014) 88-92. https://doi.org/10.1016/j.ijpharm.2014.09.044 10.1016/j.ijpharm.2014.09.044 25275937

[61] Arafat B. Wojsz M. Isreb A. Forbes R.T. Isreb M. Ahmed W. Arafat T. Alhnan M.A.. Tablet fragmentation without a disintegrant: A novel design approach for accelerating disintegration and drug release from 3D printed cellulosic tablets. European Journal of Pharmaceutical Sciences 118 (2018) 191-199. https://doi.org/10.1016/j.ejps.2018.03.019 10.1016/j.ejps.2018.03.019 29559404

[62] Sadia M. Arafat B. Ahmed W. Forbes R.T. Alhnan M.A.. Channelled tablets: An innovative approach to accelerating drug release from 3D printed tablets. Journal of controlled release 269 (2018) 355-363. https://doi.org/10.1016/j.jconrel.2017.11.022 10.1016/j.jconrel.2017.11.022 29146240

[63] Isreb A. Baj K. Wojsz M. Isreb M. Peak M. Alhnan M.A.. 3D printed oral theophylline doses with innovative ‘radiator-like’design: Impact of polyethylene oxide (PEO) molecular weight. International Journal of Pharmaceutics 564 (2019) 98-105. https://doi.org/10.1016/j.ijpharm.2019.04.017 10.1016/j.ijpharm.2019.04.017 30974194

[64] Khaled S.A. Alexander M.R. Irvine D.J. Wildman R.D. Wallace M.J. Sharpe S. Yoo J. Roberts C.J.. Extrusion 3D printing of paracetamol tablets from a single formulation with tunable release profiles through control of tablet geometry. AAPS PharmSciTech 19 (2018) 3403-3413. https://doi.org/10.1208/s12249-018-1107-z 10.1208/s12249-018-1107-z 30097806 PMC6848047

[65] Goyanes A. Martinez P.R. Buanz A. Basit A.W. Gaisford S.. Effect of geometry on drug release from 3D printed tablets. International Journal of Pharmaceutics 494 (2015) 657-663. https://doi.org/10.1016/j.ijpharm.2015.04.069 10.1016/j.ijpharm.2015.04.069 25934428

[66] Palekar S. Nukala P.K. Mishra S.M. Kipping T. Patel K.. Application of 3D printing technology and quality by design approach for development of age-appropriate pediatric formulation of baclofen. International Journal of Pharmaceutics 556 (2019) 106-116. https://doi.org/10.1016/j.ijpharm.2018.11.062 10.1016/j.ijpharm.2018.11.062 30513398

[67] Raje V. Palekar S. Banella S. Patel K.. Tunable Drug Release from Fused Deposition Modelling (FDM) 3D-Printed Tablets Fabricated Using a Novel Extrudable Polymer. Pharmaceutics 14 (2022) 2192. https://doi.org/10.3390/pharmaceutics14102192 10.3390/pharmaceutics14102192 36297626 PMC9611745

[68] Melocchi A. Briatico-Vangosa F. Uboldi M. Parietti F. Turchi M. von Zeppelin D. Maroni A. Zema L. Gazzaniga A. Zidan A.. Quality considerations on the pharmaceutical applications of fused deposition modeling 3D printing. International Journal of Pharmaceutics 592 (2021) 119901. https://doi.org/10.1016/j.ijpharm.2020.119901 10.1016/j.ijpharm.2020.119901 33010399

[69] Parikh T. Sandhu H.K. Talele T.T. Serajuddin A.. Characterization of solid dispersion of itraconazole prepared by solubilization in concentrated aqueous solutions of weak organic acids and drying. Pharmaceutical research 33 (2016) 1456-1471. https://doi.org/10.1007/s11095-016-1890-8 10.1007/s11095-016-1890-8 26951566

[70] Shah A. Serajuddin A., Supersolubilization by using nonsalt-forming Acid-Base interaction, in Amorphous Solid Dispersions, Springer 2014, p. 595-611.

[71] Singh S. Parikh T. Sandhu H.K. Shah N.H. Malick A.W. Singhal D. Serajuddin A.. Supersolubilization and amorphization of a model basic drug, haloperidol, by interaction with weak acids. Pharmaceutical research 30 (2013) 1561-1573. https://doi.org/10.1007/s11095-013-0994-7 10.1007/s11095-013-0994-7 23430485

[72] Serajuddin A.T.. Salt formation to improve drug solubility. Advanced Drug Delivery Reviews 59 (2007) 603-616. https://doi.org/10.1016/j.addr.2007.05.010 10.1016/j.addr.2007.05.010 17619064

[73] Serajuddin A.T. Jarowski C.I.. Effect of diffusion layer pH and solubility on the dissolution rate of pharmaceutical bases and their hydrochloride salts I: phenazopyridine. Journal of Pharmaceutical Sciences 74 (1985) 142-147. https://doi.org/10.1002/jps.2600740208 10.1002/jps.2600740208 3989682

[74] Parikh T. Serajuddin A.. Development of fast-dissolving amorphous solid dispersion of itraconazole by melt extrusion of its mixture with weak organic carboxylic acid and polymer. Pharmaceutical research 35 (2018) 1-10. https://doi.org/10.1007/s11095-018-2407-4 10.1007/s11095-018-2407-4 29696402

[75] Aho J. Bøtker J.P. Genina N. Edinger M. Arnfast L. Rantanen J.. Roadmap to 3D-printed oral pharmaceutical dosage forms: feedstock filament properties and characterization for fused deposition modeling. Journal of Pharmaceutical Sciences 108 (2019) 26-35. https://doi.org/10.1016/j.xphs.2018.11.012 10.1016/j.xphs.2018.11.012 30445005

[76] GUIDANCE FOR INDUSTRY - Dissolution Testing of Immediate Release Solid Oral Dosage Forms, https://www.fda.gov/media/70936/download (November 12, 2022).

[77] Dressman J.B. Amidon G.L. Reppas C. Shah V.P.. Dissolution testing as a prognostic tool for oral drug absorption: immediate release dosage forms. Pharmaceutical research 15 (1998) 11-22. https://doi.org/10.1023/A:1011984216775 10.1023/A:1011984216775 9487541

